# De l'hémoglobine SS à SF: intérêt de l'hydroxyurée dans la prise en charge de la drépanocytose chez 2 enfants congolais et revue de la literature

**DOI:** 10.11604/pamj.2015.21.124.5784

**Published:** 2015-06-15

**Authors:** Gayllord Mutoke Nkashama, Gray Kanteng A Wakamb, Augustin Mutombo Mulangu, Georges Mutoke Nkashama, Boniface Kabeya Kupa, Oscar Luboya Numbi

**Affiliations:** 1 Université de Lubumbashi, BP 1825, Département de Pédiatrie, Cliniques Universitaires de Lubumbashi, Lubumbashi, République Démocratique du Congo

**Keywords:** Drépanocytose, hydroxyurée, hémoglobine fœtale, sickle cell disease, hydroxyurea, feotale hemoglobin

## Abstract

La drépanocytose est une maladie grave par ses complications et par les difficultés liées à sa prise en charge, notamment en milieu sous-équipé. Les auteurs rapportent l'effet bénéfique de l'hydroxyurée dans la prise en charge de deux patients drépanocytaires, au prix cependant d'une surveillance hématologique rigoureuse. Une revue de la littérature étaye par ailleurs les modalités d'administration de ce médicament et les perspectives ultérieures d'un tel traitement dans la drépanocytose.

## Introduction

Les traitements symptomatiques (antibiotiques et transfusion surtout) avaient déjà permis d'améliorer nettement l'espérance de vie des patients atteints de syndrome drépanocytaire majeur. L′hydroxyurée est le premier traitement fondé sur la physiopathologie propre de la maladie qui diminue la fréquence des crises douloureuses chez la plupart des patients et allonge leur espérance de vie [1, 2]. Au début de son utilisation chez les patients drépanocytaires, le bénéfice de l'hydroxyurée était attribué seulement à la réactivation de la synthèse d'hémoglobine fœtale. Cet effet bénéfique s'est plus tard aussi avéré lié à de multiples autres processus tels que la réduction de l'adhésion excessive des érythrocytes à l'endothélium et sans doute la leucopénie relative induite par le traitement. Les objectifs de cette présentation clinique sont de: décrire l’évolution de la drépanocytose sous traitement à l'hydroxyurée chez deux patients congolais; mettre en évidence les perspectives d'un tel traitement tel que présenté dans la littérature.

## Patient et observation

**Observation 1**: Il s'agissait d'un patient de sexe masculin âgé de 4 ans et pesant 14 kg, soit un déficit pondéral chiffré à 13%. Il est venu consulter pour la première fois pour tuméfaction du dos de la main droite depuis quelques heures et fièvre depuis 10jours. Antérieurement, il avait reçu un traitement fait de quinine en perfusion et gentamycine injectable. Comme antécédents, il a été mis en évidence 2 transfusions à l’âge de 6 mois et à l’âge de 15 mois; avec une notion de syndrome pied-mains. A l'examen physique, l’état général était marqué par l'asthénie physique; l'enfant était non fébrile avec un crane en tour. Ces conjonctives palpébrales étaient colorées et les bulbaires sub-ictériques. Par ailleurs un souffle tubaire au sommet du poumon droit était notable, avec une splénomégalie type1 selon Hacket; une tuméfaction douloureuse du dos de la main droite avec chaleur locale. Un diagnostic de crise vaso-occlusive et de pneumonie droite chez un drépanocytaire probable est alors posé. L’électrophorèse de l'hémoglobine réalisée a révélé: sujet homozygote SS pas de trace d'hemoglobine fœtale et l'hemogramme: hemoglobine a 9gr% globules blancs: 10100/mm fl: neutro 60% lympho 40% la vs10mm/h. Au bout de 7 jours de traitement fait d'hydradation d'antalgique d'antibiotiques plus acide folique le malade a bien evolué. Ensuite le patient a beneficié d'un traitement aux vaccins spécifiques et a été soumis sous hydroxyurée a raison de 1cés de 500mg 3 fois par semaine avec contrôle mensuel de l'hemmograme (Hb,GB,FL et Plaquettes). Au bout de 5 ans de suivi sous hydroxyurée on a noté une attenuation des crises et son électrophorèse d’ hemoglobine s'est presentée comme suit: hbF: 27.1% hbS: 68.9% et hbA2: 4%.

**Observation 2**: Malade x de sexe f agé de 9 ans pesant 22kg soit un deficit chiffre à 18.5% venu consulter pour la première fois en 2003 pour fièvre depuis 4 jours et douleurs abdominales. traitement recu quinine ampicilline. Dans ses antécédents on note qu'elle est drépanocytaire homozygote depuis x année (électrophorèse déjà realisée sujet SS) plusieurs fois transfusée et qu'elle a connu un avc avec sequelles motrices (hemiparésie droite, dysarthrie et troubles visuels) il y a 3 ans. A l'examen physique on note un état général marqué par l'asthenie physique signes vitaux dans les normes conjonctives palpébrales colorées et bulbaires anictériques. L'examen du cœur et des poumons n'a rien révelé. L'abdomen est non ballonné avec sensibilité marquée à la région hypogastrique sans organomegaliepalpée. Un diagnostic de crises vaso-occlusives et d'infection urinaire sont retenus (résultat labo a compléter…) et un traitement fait d'hydratation d'antibiotique plus acide folique est instauré. Apres stabilisation de l’état général un traitement à base d'hydroxyurée est debuté. Au bout de 2 ans de suivi clinique et paraclinique (hemogramme: Hb,GB,FL et Plaquettes) un contrôle d’électrophorèse est realisé dont voici les resultats: hbF: 24% hbS: 66%.

## Discussion

Des faisceaux d´arguments montrent que l´HbF exerce un effet atténuateur sur la sévérité de la drépanocytose. Les sujets drépanocytaires ayant conjointement une persistance héréditaire de l´HbF et les patients de certaines ethnies (Arabie Saoudite, Inde) ayant un taux élevé d´HbF ont des maladies moins sévères [3, 4]. L´apparition de complications spécifiques telles que le syndrome pied-main, le syndrome thoracique aigu ou les crises vaso-occlusives sont contemporaines de la cinétique de décroissance du taux d´HbF dans lespremiers mois de vie. Chez l´enfant, il existe des relations très significatives entre un taux bas d´HbF et la survenue des crises douloureuses et les syndromes thoraciques aigus [3, 4]. En 1968 àkinshasa Michaux et al rapportent un cas de diagnostic tardif de drépanocytosegrâce à la persistance de l'hémoglobine fœtale [5]. L'observation que la sévérité de la maladie drépanocytaireétait inversement corrélée au taux d'HbF a conduit à tenter de réactiver la synthèse de l'Hb, l'hydroxyurée paraissant la molécule réactivatrice de la synthèse d'HbF la mieux tolérée [6]. Plusieurs études à travers le monde s'y sont penchées comme celle de F. Mellouli et al réalisée en Tunisie en 2007 qui rapporte une augmentation significative de l hemoglobine fœtale de 3 à 30% après usage de l'hydroxyurée pendant 6 ans et 9 mois [7]. En1996 une étude belge menée en Afrique centrale chez 22 enfants âgés en moyenne de 8 ans, traités 6 mois par 20 mg/kg/j d´hydroxyurée a révélé une réduction significative du nombre de jours d´hospitalisation pour crise douloureuse [8]. Il est apparu assez vite que les patients traités par Hydroxyurée éprouvaient un bénéfice clinique antérieur à l´augmentation de leur taux d´HbF, parfois dès quelques semaines de traitement, alors que l´augmentation du taux d´HbF s´observe en règle après le 3e mois.

L´importance de l´amélioration clinique n´est d´autre part pas corrélée avec l´élévation du taux d´HbF [9]. Ces constatations étayent l´hypothèse que, même si la réactivation de la synthèse de l´HbF joue un rôle important dans l´action de l´Hydroxyurée, l´amélioration de la maladie est aussi liée à d´autres facteurs dont la hiérarchie reste àpréciser. Au fil des ans d'autres mécanismes d'action ont été suggérés: la réduction de l'adhésion excessive des réticulocytes drépanocytaires à l'endothélium, la diminution des leucocytes, la modulation des processus inflammatoires: effets cellulaires (diminution des cellules denses), l´induction du monoxyde d'azote (NO). Le fait de faire plus de 3 crises douloureuses par an et ou plus de 2 syndromes thoraciques aigus sont maintenant des indications bien établies de l´ hydroxyurée [6]. Cependant certaines nouvelles indications restent à discuter (telle la prévention des AVC chez les patients asymptomatiques révélés « à risque » par un doppler transcrânien). Les tolérances à court et à moyen termes sont bonnes sous surveillance clinique et paraclinique (hématologique). Des épisodes de myélotoxicité (neutropénies et/ou thrombopénies) modérés le plus souvent s´amendent à la diminution des doses parfois après un arrêt transitoire du traitement et la croissance staturo-pondérale, l´acquisition de signes pubertaires ne sont pas modifiées nous rapportent deux études américaines réalisées respectivement en 1999 et 2001 [10, 11]. Des résultats analogues émanent d´une autre étude américaine de 1999 qui relate 2 ans de surveillance chez 24 enfants [12]. L´âge reste un critère important en ce qui concerne le début du traitement. Des données récentes font préférer d'attendre l´âge de 2 ans pour commencer un traitement par hydroxyurée chez un enfant drépanocytaire [13]. La principale question qui reste en suspens est la tolérance à long terme. Les risques mutagènes et cancérogènes craints initialement paraissent extrêmement faibles. En revanche on ignore si le traitement à l´hydroxyurée a des conséquences négatives sur la fertilité ultérieure de garçons qui auraient été traités très jeune et pendant très longtemps, d'autant plus que la drépanocytose peut déjà par elle-même induire des modifications de la qualité du sperme [4]. Une publication de 2007 rapporte 2 cas d'oligo/azoospermies non réversibles à l'arrêt du traitement [14]. Ces incertitudes nous amènent à penser que l'hydroxyurée doit pour l'instant être réservée aux formes les plus symptomatiques de la maladie.

## Conclusion

L'hydroxyurée est capable de transformer la qualité de vie de certains patients, comme le prouve le cas cliniques décrits dans cette série. Le bénéfice est obtenu par une réduction sensible des crises douloureuses chez les patients drépanocytaires. Sa toxicité à court et à moyen terme est modeste, quant à sa toxicité à long terme elle reste encore incertaine ce qui justifie son usage dans les formes sévères de drépanocytose.
